# Acetic acid enabled nuclear contrast enhancement in epi-mode quantitative phase imaging

**DOI:** 10.1117/1.JBO.30.2.026501

**Published:** 2025-02-04

**Authors:** Zhe Guang, Amunet Jacobs, Paloma Casteleiro Costa, Zhenmin Li, Francisco E. Robles

**Affiliations:** a Emory University, Georgia Institute of Technology, Wallace H. Coulter Department of Biomedical Engineering, Atlanta, Georgia, United States; b University of Kentucky, College of Medicine, Lexington, Kentucky, United States; c Georgia Institute of Technology, School of Electrical and Computer Engineering, Atlanta, Georgia, United States

**Keywords:** quantitative phase imaging, acetic acid, nuclear contrast, microscopy

## Abstract

**Significance:**

The acetowhitening effect of acetic acid (AA) enhances light scattering of cell nuclei, an effect that has been widely leveraged to facilitate tissue inspection for (pre)cancerous lesions. Here, we show that a concomitant effect of acetowhitening—changes in refractive index composition—yields nuclear contrast enhancement in quantitative phase imaging (QPI) of thick tissue samples.

**Aim:**

We aim to explore how changes in refractive index composition during acetowhitening can be captured through a novel epi-mode 3D QPI technique called quantitative oblique back-illumination microscopy (qOBM). We also aim to demonstrate the potential of using a machine learning-based approach to convert qOBM images of fresh tissues into virtually AA-stained images.

**Approach:**

We implemented qOBM, an imaging technique that allows for epi-mode 3D QPI to observe phase changes induced by AA in thick tissue samples. We focus on detecting nuclear contrast changes caused by AA in mouse brain samples. As a proof of concept, we also applied a Cycle-GAN algorithm to convert the acquired qOBM images into virtually AA-stained images, simulating the effect of AA staining.

**Results:**

Our findings demonstrate that AA-induced acetowhitening leads to significant nuclear contrast enhancement in qOBM images of thick tissue samples. In addition, the Cycle-GAN algorithm successfully converted qOBM images into virtually AA-stained images, further facilitating the nuclear enhancement process without any physical stains.

**Conclusions:**

We show that the acetowhitening effect of acetic acid induces changes in refractive index composition that significantly enhance nuclear contrast in QPI. The application of qOBM with AA, along with the use of a Cycle-GAN algorithm to virtually stain tissues, highlights the potential of this approach for advancing label-free and slide-free, *ex vivo*, and *in vivo* histology.

## 1 Introduction

Quantitative phase imaging (QPI) enables detailed analysis of subcellular structures, refractive index variations, and dynamics, allowing for real-time imaging of live cells with negligible phototoxicity. It is thus an essential approach for understanding cellular processes and dynamic activities over extended periods of time, providing insights into cell behavior, disease progression, and responses to therapeutic interventions or external stimuli.^1^ Among the various embodiments of QPI, quantitative oblique back-illumination microscopy (qOBM) uniquely enables QPI in scattering samples using epi-illumination,^2^^,^^3^ providing non-invasive observations of cell morphology,^4^^–^^6^ and dynamics^7^^,^^8^ in thick tissues or complex scattering structures, which are not accessible in a transmission optical configuration that is used in other QPI embodiments. One exciting clinical application of qOBM is in neurosurgery where the approach can potentially be applied for intraoperative tumor margin identification *in vivo*.^9^^–^^11^ However, similar to other QPI-based methods, qOBM has limited contrast for cell nuclei, which is a key histological feature to assess disease. Currently, optical stains and fluorescent agents are widely employed to enhance nuclear contrast in microscopy. Fluorophores such as DAPI^12^ or Hoechst^13^ selectively bind to DNA, providing clear visualization of cell nuclei. Immunofluorescence techniques that utilize fluorescently labeled antibodies against nuclear proteins are also commonly applied.^14^ Although these methods significantly enhance nuclear contrast, they come with several limitations: (1) the staining process can be invasive, potentially altering cell local environments with possible cytotoxic effects; (2) fluorophores are susceptible to photobleaching; (3) there may be requirements for specialized equipment and multi-color imaging which pose additional technical challenges; and (4) such agents, for the most part, cannot be used clinically *in vivo*. A fast and easy way to enhance nuclear phase contrast, *in vivo* and *in situ*, is therefore highly preferred.

Acetic acid (AA) has been widely adopted as an *in vivo* agent to increase nuclear visibility^15^^–^^17^ for diagnosing various diseases, such as cervical cancer,^18^^–^^20^ oral mucosal neoplasia,^21^^,^^22^ skin or epithelial diseases,^23^^,^^24^ and solid tumors.^25^ AA causes denaturation of proteins (histone deacetylation and cytokeratin polymerization), leading to condensed chromatin in cell nuclei.^17^ This process alters the refractive index of tissues, which also results in changes in scattering. This leads to a visual whitening effect termed “acetowhitening,” which can highlight precancerous or abnormal regions that may require further investigation, biopsy, or closer monitoring. Many imaging techniques, such as confocal microscopy,^26^^,^^27^ optical coherence tomography,^24^ and diffuse optical microscopy,^28^ have leveraged AA acetowhitening for nuclear enhancement and disease detection. Particularly, in reflectance confocal microscopy, near real-time nuclear segmentation has been achieved on amelanotic cervical tissues,^29^ and AA staining has been studied in comparison to histopathological analysis for potential use to guide Mohs micrographic surgeries.^30^

More recently, with the emergence of deep learning technologies, various computational algorithms have been proposed to facilitate histological analysis, offering the possibility to enhance nuclear contrast by virtual staining without applying physical stains or fluorescent labels.^31^ Such methods are particularly valuable in live-cell *in vivo* imaging where physical staining is not possible, practical, or may interfere with inherent cell properties. For example, Li et al. recently reported on a generative adversarial network (GAN)-based algorithm to virtually stain reflectance confocal microscopy skin images *in vivo*, using AA-enhanced nuclear scattering and subsequent pseudo-hematoxylin and eosin (H&E) colorization.^32^ These prior works indicate that AA-enhanced nuclear contrast may also be a valuable tool for QPI, particularly using qOBM given its unique ability to image thick tissues.

In this paper, we report on AA-enhanced nuclear phase contrast in thick tissues observed by qOBM. As a proof of concept, we also demonstrate virtual AA staining of fresh tissue samples using a cycle-consistent generative adversarial network (Cycle-GAN) algorithm,^33^ which could have important implications for facilitating *in vivo* disease diagnosis with qOBM.

## 2 Imaging Setup and Principles

### 2.1 Experimental Apparatus and Image Reconstruction

**Fig. 1:**
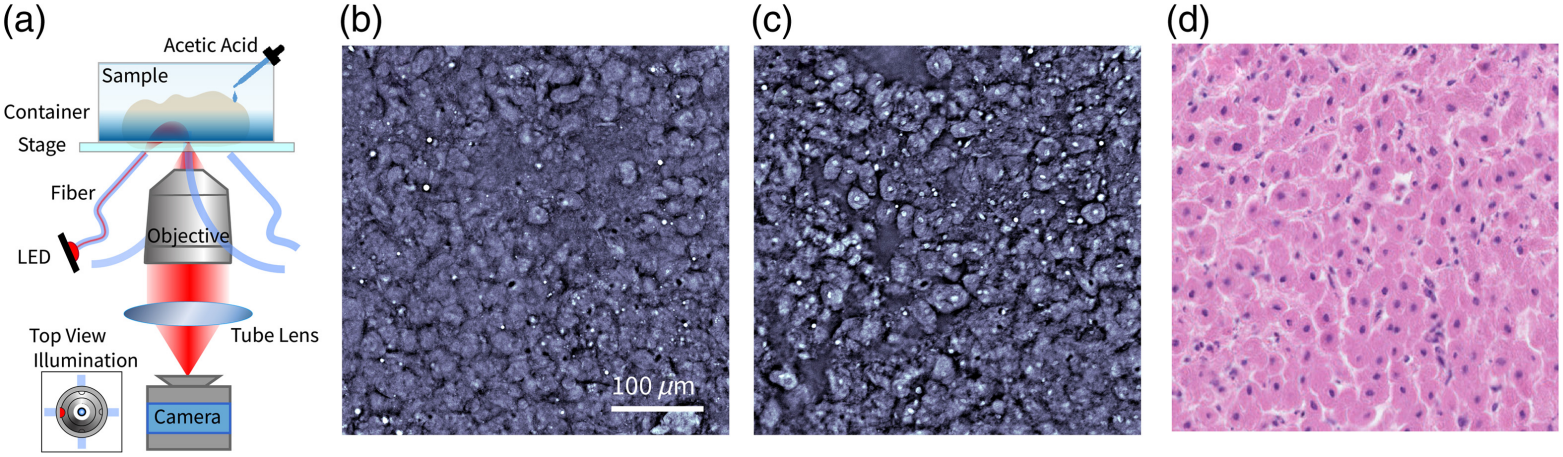
(a) Experimental setup of qOBM. (b) qOBM image of freshly excised bovine liver. (c) qOBM image of the bovine liver after applying AA stain. (d) H&E-stained microscopic image of the bovine liver.

To explore the impact of AA acetowhitening on nuclear phase contrast, we use an inverted qOBM microscope system. Figure 1(a) shows the qOBM experimental setup. The figure depicts a thick sample that is immersed in AA solution and placed in a container atop a motor stage. A 0.6 NA 40× (Nikon ELWD 40XC) or 0.7 NA 60× (Nikon ELWD 60XC) objective is used to image the sample onto a camera (sCMOS pco.edge 4.2 LT) via a 180-mm tube lens. Four 720-nm LEDs are coupled to multimode fibers to illuminate the sample obliquely (45 deg) from below. The four fibers form two pairs of opposing illumination. Image processing to obtain a quantitative phase has been described in our group’s earlier work.^2^^,^^3^ In short, images with opposing illumination are subtracted and then normalized by their sum to obtain two orthogonal differential phase contrast (DPC) images. Then, the two DPC images are deconvolved using the system’s optical transfer function to obtain a quantitative phase image. We note that similar to other computational phase imaging methods, this DPC reconstruction has relatively low sensitivity to very low-frequency structures;^34^ nevertheless, the quantitative fidelity of qOBM has been evaluated and verified using a photolithographic phase targe with nanometer structures, micron-sized beads, and red blood cells.^2^

### 2.2 Acetic Acid Staining Effect

With this qOBM system, we observe nuclear contrast changes in various samples due to the AA staining effect. All analyzed samples are commercially available or discarded from unrelated approved experiments. Fresh tissues are first imaged without AA, and then, AA is applied with a Pipette without moving the sample in the container, as shown in Fig. 1(a). During the process, no additional sample preparation is needed. Figures 1(b) and 1(c) show qOBM images of a fresh bovine liver sample before and after applying AA (25% vol. concentration solution, for 15 min) to enhance the nuclear contrast. The relatively high concentration ensures that AA penetrates deep into the thick tissue (for reference, a 50% AA solution was used in confocal microscopy to study skin tumors^32^). Note that AA concentrations ranging from 6% to 50% were tested, as were staining times from 5 to 20 min. From these tests, we identified that staining for 15 min with an AA concentration of 25% was optimal for the nuclear enhancement of thick tissues with qOBM. Compared with fresh liver in Fig. 1(b), AA-stained liver tissue in Fig. 1(c) shows nuclei with higher phase values and hence higher refractive indices. Note that some high-phase contrast, round-shaped objects are found outside of the cells in both the fresh and AA-stained images—these are believed to be lipid droplets. Figure 1(d) shows a representative H&E-stained slide of the same bovine liver sample (different region). Even though the H&E preparation procedures alter the tissue structure and prevent the same exact locations from being imaged between qOBM and H&E, the nuclear morphology in the AA-stained qOBM image indeed shows good agreement with the H&E image. This indicates that AA staining may serve as a good candidate to improve nuclear contrast for qOBM.

## 3 Results and Discussion

### 3.1 Acetic Acid Staining of Fresh Mouse Brain

**Fig. 2:**
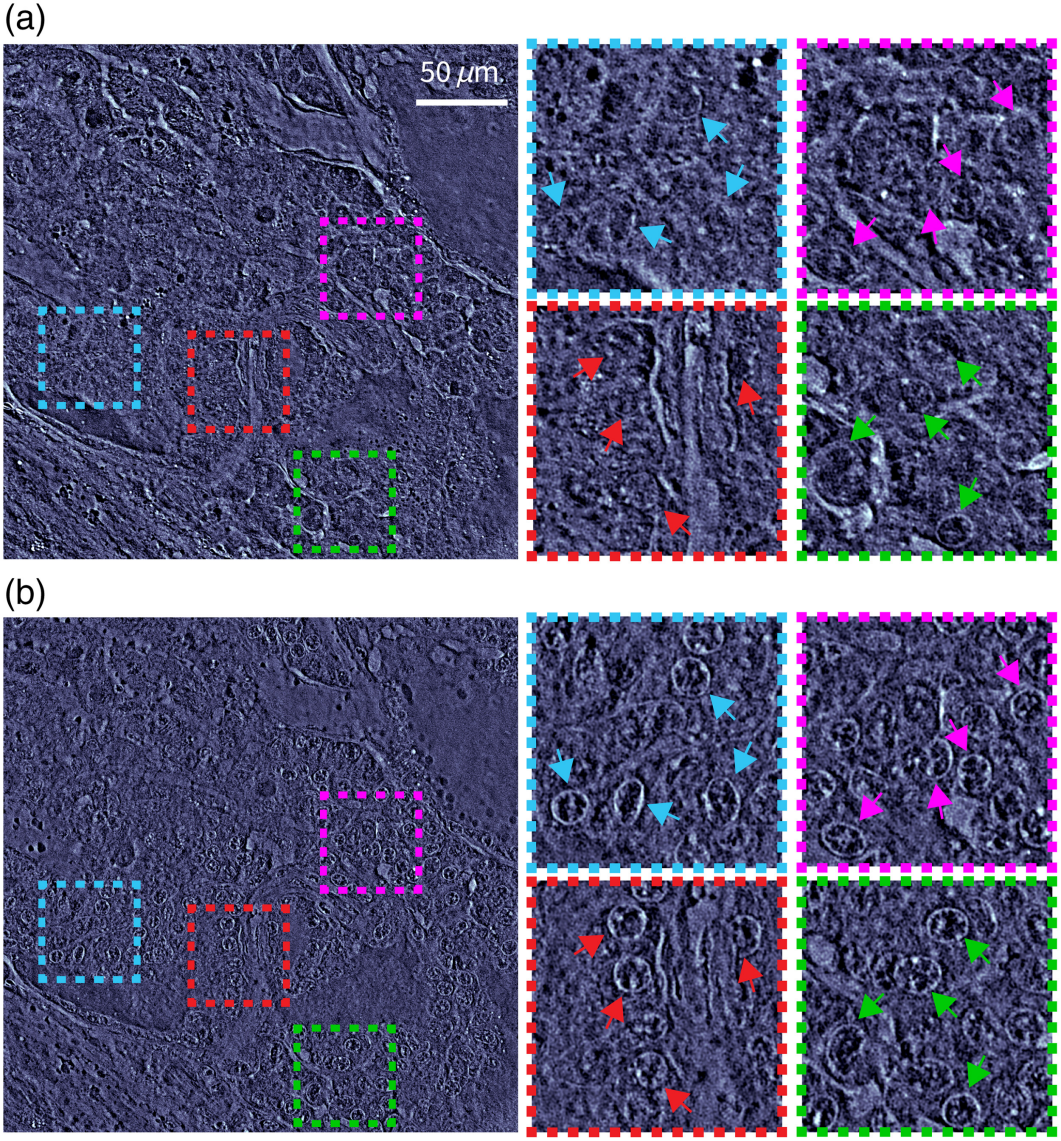
Fresh mouse brain tissue changes due to AA staining. (a) qOBM image before applying AA. (b) qOBM image of approximately the same region after applying AA. Dashed color insets show regions with nuclear contrast enhancement indicated by color arrows (Video 1, MP4, 27.3 MB [URL: https://doi.org/10.1117/1.JBO.30.2.026501.s1]; Video 2, MP4, 22.1 MB [URL: https://doi.org/10.1117/1.JBO.30.2.026501.s2]; Video 3, MP4, 57.4 MB [URL: https://doi.org/10.1117/1.JBO.30.2.026501.s3]; Video 4, MP4, 60.2 MB [URL: https://doi.org/10.1117/1.JBO.30.2.026501.s4]).

Figure 2 shows a different example using a fresh mouse brain. Figures 2(a) and 2(b) show the same mouse brain region before and after applying AA, respectively. As the figure shows, AA significantly alters the nuclear visibility—for example, cell nuclei before and after staining are demarcated by color arrows in the insets. Similar to the liver tissue, these samples are stained in AA for 15 min, and observations after 15 min (up to 30 min) showed that tissue nuclear visibility remained the same, indicating a thorough staining. Further, to visualize AA staining in a volume, we also took Z scans of the mouse brain samples, with qOBM images taken every ∼1.5  μm steps in depth, starting from the tissue surface and going deeper into the tissue (see Video 1 for the fresh brain and Videos 2–4 for AA-stained brain tissues). The nuclear contrast enhancement is clearly visible in the entire imaged volume. Additional sub-cellular and extracellular structures can also be observed, although such structures appear more homogeneous after AA staining. We note that qOBM typically has a penetration depth of ∼200  μm, but the increased scattering resulting from AA-staining reduces it by a factor of about 40%.

**Fig. 3:**
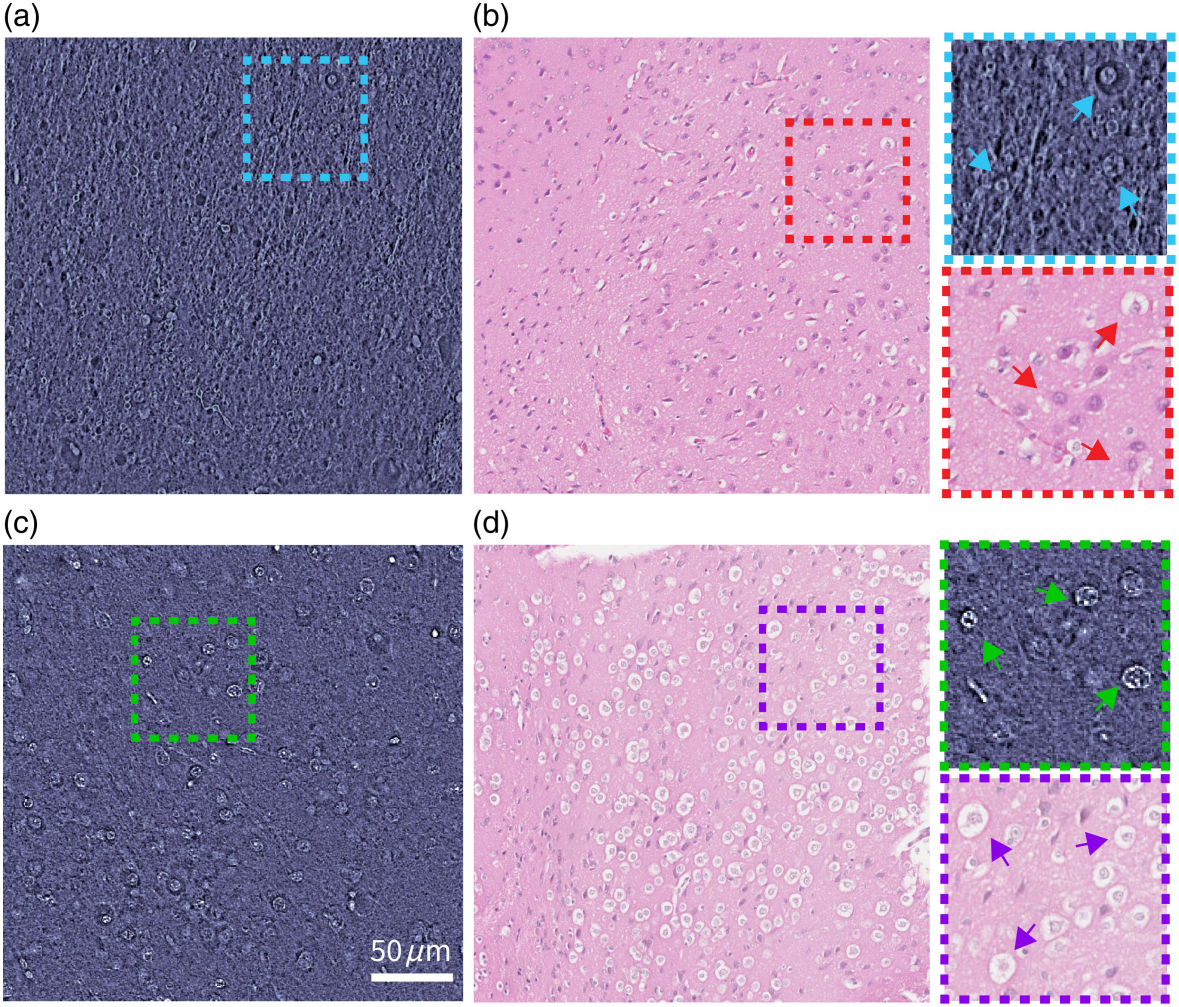
Histological effects of AA staining. Panels (a) and (b) show a qOBM image and an H&E-stained slide image, respectively, of a fresh mouse brain tissue without AA staining. Panels (c) and (d) show a qOBM and a H&E image, respectively, with AA staining. Insets highlight representative nuclear structures.

AA staining is a relatively fast and effective way to enhance nuclear contrast in QPI (and other optical technologies). However, adding AA as a staining agent introduces alterations to tissues: cell nuclei contract, tissues become denser, and red blood cells lyse. These changes not only make it difficult to precisely pixel match and compare images of fresh and AA-stained tissues but also produce irreversible and significant changes to the histology. Figure 3 shows the effects of AA staining on the histology of mouse brain samples. Figures 3(a) and 3(b) show the qOBM and H&E slide images, respectively, without applying AA, whereas Figs. 3(c) and 3(d) show the corresponding images after applying AA. Note that similar regions are selected for comparison. As clearly seen, AA staining results in hollow cell structures with condensed nuclei in H&E. This alteration resulting from the AA stain may lead to confusion or misinterpretation in H&E histopathology analysis, which could have deleterious clinical implications.

### 3.2 Cycle-GAN Algorithm for Virtual Staining

As a potential alternative to solve this problem, we implement an algorithm to enable virtual AA staining. Such an approach can avoid issues with damaged tissues and distorted H&E histology while still benefiting from the nuclear phase contrast enhancement. With the exponential growth of deep learning technologies, it is possible to utilize existing pipelines to aid the image translation (this includes a similar pipeline recently proposed for reflectance confocal microscopy).^11^^,^^31^^,^^32^^,^^35^ Note that pixel-to-pixel mapping of AA-stained and unstained qOBM phase images is not possible, because the tissue (particularly the brain) undergoes noticeable deformation as the sample is stained, even if the field of view is constant and the sample remains immobile. Thus, unsupervised methods for image translation are necessary.

**Fig. 4:**
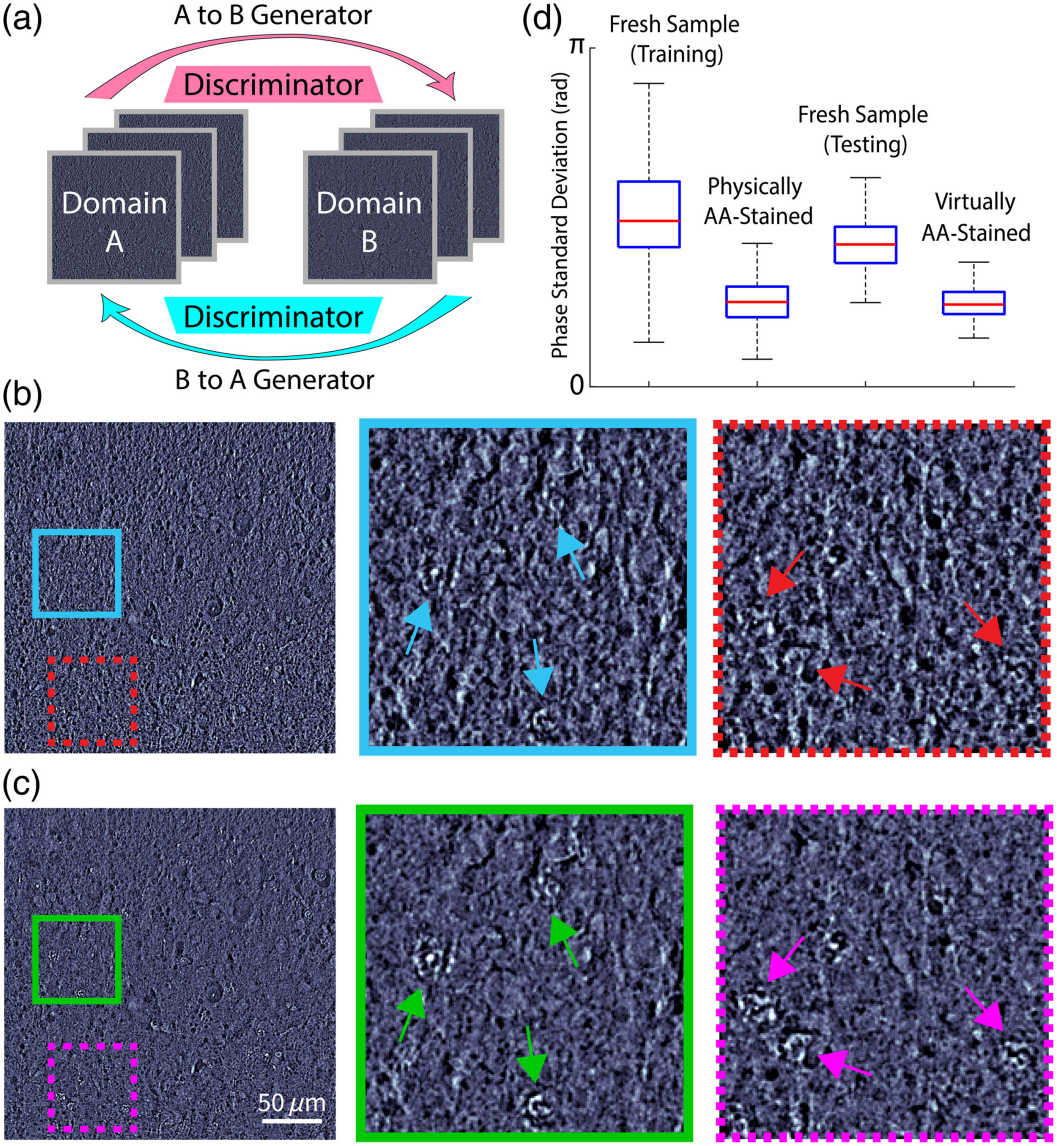
Cycle-GAN virtual staining results. (a) Cycle-GAN algorithm architecture. Representative input (b) and output (c) images of Cycle-GAN, demonstrating virtual AA staining with nuclear contrast enhancement. Insets show nuclei enhancement (arrows). (d) Distribution of phase standard deviations.

For this proof of concept, we use a cycle-consistent generative adversarial network (cycle-GAN)^33^ which is an unsupervised framework that comprises two major components: a generator and a discriminator. The generator maps images from one domain to another, whereas the discriminator attempts to distinguish real and fake (generated) images in the targeted domain. The Cycle-GAN architecture and image processing results are shown in Fig. 4. Figure 4(a) shows the Cycle-GAN from “Domain A”—qOBM images of the fresh brain—to “Domain B”—qOBM images of physically AA stained brain. Once trained, the “A to B generator” output can be treated as a virtual AA stain for qOBM with enhanced nuclear details. Our dataset is composed of 2466 fresh brain images and 1305 AA-stained brain images (image size 512-by-512-pixel) from 10 healthy mice and a split ratio of 75:25 for training and testing. The network training uses brain images from the surface up to ∼150  μm into the tissue. The model is trained on an NVidia RTX 2080 Ti GPU with 200 epochs, which takes about 9 h to complete.

Figures 4(b) and 4(c) show representative input and output images of the Cycle-GAN virtual AA stain. Compared with the images of the fresh brain [Fig. 4(b)], the virtually AA-stained image [Fig. 4(c)] presents cell nuclear details with higher phase contrast while homogenizing the phase values of surrounding tissue structures, thus providing better nuclear visibility. These types of features match well with those of the physically AA-stained samples, as shown in Fig. 3(c). The insets of Figs. 4(b) and 4(c) show cell nuclei before and after virtual AA staining. Similar to the physical stain, virtual AA staining also presents enhanced cell nuclei, but now with no physical or biochemical alteration to the fresh samples, which is beneficial for downstream histological analysis with H&E, for example. Note that, qualitatively, the degree of nuclear contrast enhancement with the virtual stain is not quite as striking as that of the physical AA stain. We attribute this to the limited data set in this proof of concept, and we expect improvements with a larger data set and more extensive training. Moreover, with appropriate training data sets, a similar network architecture can be applied to other sample types (such as the bovine liver in Fig. 1).

To provide a quantitative comparison for this proof of concept, Fig. 4(d) shows the standard deviations of qOBM phase values among four groups: fresh samples (used in training data), physically AA stained samples (AA training data), fresh samples (used for testing), and virtually AA stained samples from the test set. We note that, for fresh samples, the training and testing datasets show some differences in standard deviations, which is a result of the smaller set of images used in testing. Nevertheless, physically and virtually AA-stained images show a quite consistent phase standard deviations (not statistically different; two-sample t-test p-value: 0.6442) and both are quite different from the fresh samples (p-values <0.0001). This indicates that the translated virtually AA-stained qOBM images share a similar phase distribution to those that are physically stained. This also shows, as previously observed, that the AA-staining makes qOBM images have higher phase contrast of cell nuclei while smoothing/homogenizing non-nuclear regions for an overall decrease in the variance of phase values.

## 4 Conclusions

In conclusion, we investigated how AA staining affects quantitative phase images of fresh thick tissues using qOBM. We showed that AA effectively highlights cell nuclei while homogenizing non-nuclear structures. The resulting architecture showed good qualitative agreement with standard H&E images (using prepared slides of the same samples). As a contrast agent, AA stains the tissue relatively quickly, particularly compared with the H&E staining process which usually takes hours to complete. In addition, because AA is a clear solution, the AA staining procedure is straightforward and simply requires immersing the sample into AA. The samples can be imaged while immersed in the solution and no washing/rinsing is necessary, which avoids handling errors. However, we find that physical AA staining—using the concentration and duration necessary for nuclear enhancement with phase contrast—is incompatible with conventional histology procedures, including H&E, as the histology is severely altered. To solve this issue, we conducted a proof of concept experiment where qOBM images of fresh tissue were virtually stained using a Cycle-GAN algorithm. Results are encouraging, showing that nuclear phase contrast, in images of fresh samples, can be enhanced without physically applying AA. This can help identify the nuclei in QPI/qOBM without interfering with conventional H&E histology. Moreover, we recently demonstrated high-fidelity image translation from qOBM directly to H&E.^11^ By better demarcating cell nuclei, an intermediate virtual AA stain step could potentially further enhance the image translation fidelity from qOBM phase to virtual H&E. This pipeline would be similar to those recently developed for reflectance confocal microscopy.^32^

There are some caveats to using AA as a stain, however. AA staining (or acetowhitening) has so far mostly been used as a qualitative measure in other studies and its effect depends on various factors, including sample type, tissue depth, staining time, and solution concentration, which requires caution and more thorough investigations. Also, other high refraction index structures in the tissue, such as lipid droplets, may confuse the interpretation of the AA staining results. Finally, Cycle-GAN can potentially introduce artifacts or hallucinate features in medical images, which may result in visually plausible but clinically inaccurate representations.^36^ We recently reported on a virtual H&E staining algorithm using qOBM phase images which used the same Cycle-GAN architecture as described here.^11^ Similar to our prior work, the virtual acetic acid stain presented here may also have potential issues with differentiating cell nuclear structures from other high refraction index structures in tissue, such as vessel walls and red blood cells, which may also confuse the interpretation of the AA staining results.

Nuclear enhancement is also commonly achieved using fluorescence, although for clinical applications, it is mostly applied *in vitro* or *ex vivo* as fluorescent agents, unlike AA, are not commonly used in humans *in vivo*. Recently, neural network models have been developed to translate label-free phase images to virtual fluorescence stains of cell nuclei and other landmark organelles.^37^ However, these efforts have been restricted to systems that operate in transmission and are thus limited to thin samples. Nuclear enhancement for unlabeled thick tissues for in vivo applications, to our knowledge, has primarily leveraged AA^32^ and/or direct unpaired/unsupervised virtual staining methods.^11^ Future work will focus on alternative pipelines for nuclear enhancement potentially leveraging fluorescence. Nevertheless, together with a Cycle-GAN virtual staining, AA provides a fast and easy choice for nuclear phase contrast enhancement and staining in QPI and qOBM that can aid in future *in vivo* and *ex vivo* histopathology analysis.

## Supplementary Material









## Data Availability

Data and code underlying the results presented in this paper are not publicly available at this time but may be obtained from the authors upon reasonable request.
